# A cross-sectional study of tuberculosis drug resistance among previously treated patients in a tertiary hospital in Accra, Ghana: public health implications of standardized regimens

**DOI:** 10.1186/s12879-018-3053-5

**Published:** 2018-04-02

**Authors:** Audrey Forson, Awewura Kwara, Samuel Kudzawu, Michael Omari, Jacob Otu, Florian Gehre, Bouke de Jong, Martin Antonio

**Affiliations:** 1 University of Ghana School of Medicine and Dentistry, Accra, Ghana; 2 Korle-Bu Teaching Hospital, Accra, Ghana; 3 University of Florida, College of Medicine, Gainesville, Florida USA; 4 Department of Chest Diseases, Korle-Bu Teaching Hospital, Accra, Ghana; 5 Medical Research Council Unit, Banjul, The Gambia; 6 Institute of Tropical Medicine, Antwerp, Belgium; 7 Microbiology and Infection Unit, Warwick Medical School, University of Warwick, Coventry, UK; 8 Faculty of Infectious and Tropical Diseases, London School of Hygiene & Tropical Medicine, London, UK

**Keywords:** Anti-tuberculosis drug resistance, MDR-TB, Pre-XDR-TB, Previously treated, Ghana

## Abstract

**Background:**

*Mycobacterium tuberculosis* drug resistance is a major challenge to the use of standardized regimens for tuberculosis (TB) therapy, especially among previously treated patients. We aimed to investigate the frequency and pattern of drug resistance among previously treated patients with smear-positive pulmonary tuberculosis at the Korle-Bu Teaching Hospital Chest Clinic, Accra.

**Methods:**

This was a cross-sectional survey of mycobacterial isolates from previously treated patients referred to the Chest Clinic Laboratory between October 2010 and October 2013. The Bactec MGIT 960 system for mycobactrerial culture and drug sensitivity testing (DST) was used for sputum culture of AFB smear-positive patients with relapse, treatment failure, failure of smear conversion, or default. Descriptive statistics were used to summarize patient characteristics, and frequency and patterns of drug resistance.

**Results:**

A total of 112 isolates were studied out of 155 from previously treated patients. Twenty contaminated (12.9%) and 23 non-viable isolates (14.8%) were excluded. Of the 112 studied isolates, 53 (47.3%) were pan-sensitive to all first-line drugs tested Any resistance (mono and poly resistance) to isoniazid was found in 44 isolates (39.3%) and any resistance to streptomycin in 43 (38.4%). Thirty-one (27.7%) were MDR-TB. Eleven (35.5%) out of 31 MDR-TB isolates were pre-XDR. MDR-TB isolates were more likely than non-MDR isolates to have streptomycin and ethambutol resistance.

**Conclusions:**

The main findings of this study were the high prevalence of MDR-TB and streptomycin resistance among previously treated TB patients, as well as a high prevalence of pre-XDR-TB among the MDR-TB patients, which suggest that first-line and second-line DST is essential to aid the design of effective regimens for these groups of patients in Ghana.

## Background

The use of standardized regimens for previously treated tuberculosis (TB) patients is a concern as inadequate therapy in the presence of pre-existing Mycobacterium tuberculosis drug resistance could lead to amplification and spread of resistance through selective pressure, increasing the risk of multi-drug resistant (MDR), and extensively drug resistant (XDR) tuberculosis [1–3]. Previously treated TB patients in areas where drug susceptibility testing (DST) is not routinely available who fail or relapse on the first-line regimen (isoniazid [H], rifampicin [R], pyrazinamide [Z], and ethambutol [E]) are given an empiric standardized retreatment regimen consisting of 2 months of HRZE plus streptomycin (S), 1 month of HRZE and 5 months HRE (2SHRZE/1HRZE/5HRE), still using first-line drugs as per current WHO guidelines [4]. Studies in other African and low-income countries have shown that there is cause for concern as treatment outcomes of the retreatment regimen show success rates of only 50–60%, worse in Category I treatment failures and smear positive patients who have relapsed [5–7]. A recent study in Uganda looking at long term mortality in patients following the retreatment regimen, with a median follow up interval of 3 years, found majority (62%) of patients had died, and some of the living still had active smear positive TB [8].

Patients with MDR-TB in Ghana, like in other TB endemic areas where DST for second-line drugs is not available, are prescribed a standardized treatment regimen. In 2015 a national review of the standardized MDR-TB regimen made capreomycin (Cm) the preferred choice of ‘injectable’ because of concern about increased renal complications with kanamycin (Km). Prior to this they were given kanamycin, except for patients over 60 years old or those at risk of impaired kidney function who were given capreomycin. Levofloxacin (Lfx) is the usual choice of fluoroquinolone as it is less expensive, but patients would rarely be switched to moxifloxacin if during treatment they showed signs of new onset or worsening kidney failure. Other drugs include prothionamide (Pto), cycloserine (Cs), and pyrazinamide (Z), with ethambutol added when first-line DST showed sensitivity of the organism to ethambutol [9]. The current national guidelines recommend an 8 month intensive phase regimen of Z-Cm(Km*)-Lfx-Pto-Cs(PAS*) and 12 months of Z-Lfx-Pto-Cs (PAS*), with kanamycin (Km*) and para-amino salicylic acid (PAS*) as alternatives to capreomycin and cycloserine respectively.

Whether these standardized regimens are appropriate is unknown and has not been studied in this country. The aim of this study was to investigate the frequency and patterns of *Mycobacterium tuberculosis* drug resistance in previously treated TB patients, to inform appropriate drug regimen selection in this group of patients.

## Methods

We carried out a cross-sectional study of previously treated patients (patients with relapse, treatment failure, failure of smear conversion, or default), who had acid-fast bacilli (AFB) smear-positive pulmonary TB and were referred to the KBTH Chest Clinic Laboratory for cultures between October 2010 and October 2013. Majority of these (84%) had been on Category I treatment only. At the time of the study, the Chest Clinic did not routinely culture the sputum of all previously treated pulmonary TB patients who had failed, relapsed or defaulted treatment. The Chest Clinic laboratory, being one of two national TB culture laboratories, receives sputum samples from treatment failure and relapse patients mostly in Greater Accra region, with few from other regions in southern Ghana. In addition, for this study we included the culture of patients who failed to convert to sputum smear negative at month 2 of the standard (Category I) regimen.

Mycobacterial culture of sputum samples was performed using the Bactec MGIT 960 system and *Mycobacterium tuberculosis* isolates confirmed by colony morphology, and with a MGIT MTBcID kit®. Isolates were prepared and stored in Tryptone soya broth with glycerol at -20 °C, until shipment on dry ice to the Bacteriology P3 Laboratory in the Medical Research Council (MRC), the Gambia. There samples were re-cultured and first-line DST performed for isoniazid, rifampicin, ethambutol and streptomycin, according to the manufacturer’s instructions for BACTEC MGIT 960 [10]. DST to pyrazinamide was not done as it is not required for routine clinical practice. MDR-TB isolates were subjected to second-line drug DST using the following drugs and cutoffs: capreomycin (2.5 μg/mL), kanamycin (5 μg/mL), ofloxacin (2 μg/mL), and ethionamide (5 μg/mL) (Sigma-Aldrich, St. Louis, Mo, USA) [11]. Descriptive statistics were used to summarize patient characteristics, frequency and pattern of drug resistance. Differences between patients with MDR and non-MDR TB were compared using Systat version 13.0 (Systat Software, Inc. San Jose, CA).

## Results

There were 177 *Mycobacterium tuberculosis* isolates shipped to the MRC, Gambia, of which 155 were from previously treated patients, the rest being newly diagnosed smear positive tuberculosis. Of the 155 study isolates from previously treated patients, 23 (14.8%) were non-viable and 20 (12.9%) contaminated isolates were excluded. The final study population consisted of 112 previously-treated patients for whom phenotypic DST data was generated. The patient features of the 43 excluded isolates did not differ significantly from those of the isolates studied, with a mean age of 39.3 years in the study group and 46.8 years in those excluded and males forming 75.9% and 83.7% respectively. The breakdown of the patients included in the study and those excluded by prior treatment status is shown in Table 1. There was no significant difference between them. Of the 112 patients studied the majority (71.4%) were presenting for the start of Category II (retreatment) regimen after treatment failure or relapse from a prior Category I treatment, while 10 on Category I treatment (8.9%) had failed to convert to smear negative after 2 months or after 1 month (in two cases). Fourteen (12.5%) had failed or relapsed on Category II at 5 months, and treatment classification data was missing for 7 patients. There were very few due to loss to follow-up (default) (Table 1).

**Table 1:** Previous first-line antituberculosis treatment status among previously treated tuberculosis patients at the Korle-Bu Teaching Hospital Chest Clinic, Accra

Treatment status	In study (*N* = 112^*^)	Excluded (*N* = 43)
Prior Cat I therapy		
Failure or Relapse after prior therapy	80 (71.4%)	35 (81.4%)
Default of prior therapy	1 (0.9%)	2 (4.5%)
AFB smear-positive after 2 months of therapy	8 (7.1%)	2 (4.5%)
Smear-positive after 1 month of therapy	2 (1.8%)	0 (0.0%)
Prior Cat II therapy		
AFB smear-positive after 5 months of therapy	14 (13.3%)	4 (9.1%)

^*^Treatment classification data was missing for 7 patients

Overall, 53 (47.3%) were susceptible to all the first-line drugs tested. Six isolates (5.4%) were pan-resistant. Any resistance to isoniazid, streptomycin, rifampicin and ethambutol was observed in 39.3%, 38.4%, 30.4% and 7.1% of the patients, respectively (Table 2). Thirty-one (27.7%) of the 112 isolates in the study were MDR.

**Table 2:** Pattern of drug resistance among previously treated tuberculosis patients at the Korle-Bu Teaching Hospital Chest Clinic, Accra

Characteristic	Number	Percent
First-line drug resistance (*n* = 112)		
Pan-sensitive	53	47.3%
Any resistance		
S	43	38.4%
H	44	39.3%
R	34	30.4%
E	8	7.1%
Mono resistance		
S	12	10.7%
H	6	5.4%
R	1	0.9%
E	0	0.0%
Poly resistance/Other		
HS	6	5.4%
RS	2	1.8%
HE	1	0.9%
(HR) MDR TB	31	27.7%
MDR TB (*n* = 31)		
HR only	7	6.3%
HRS	17	15.2%
HRE	1	0.9%
HRES	6	5.4%
Second-line drug resistance (*n* = 31)		
Pre-XDR TB^a^	11	35.5%
Ofloxacin	3	9.7%
Capreomycin	8	25.8%
Kanamycin	1^b^	3.2%
Ethionamide	10	32.3%

*S* streptomycin, *H* isoniazid, *R* rifampin, *E* ethambutol, *MDR-TB* multidrug resistant tuberculosis, *pre-XDR* pre-extensively drug resistant tuberculosis

^a^Pre-XDR TB is resistance to either a second-line injectable (kanamycin, capreomycin) or a fluoroquinolone (ofloxacin), but not both

^b^one patient had resistance to both capreomycin and kanamycin, but not ofloxacin

The MDR-TB patients were significantly more likely than those with non-MDR isolates to have streptomycin and ethambutol resistance, 74.2% and 22.6% respectively in MDR isolates compared with 24.7% and 1.2% in non MDR-TB (Table 3). Pre-extensively drug resistant (Pre-XDR) TB was found in 11 of the 31 isolates (35.5%), resistant to either ofloxacin (8 patients) or capreomycin (3 patients). One MDR isolate was resistant to both capreomycin and kanamycin, and 10 (32.3%) were resistant to ethionamide (Table 2). There were no cases of XDR-TB identified in our study population.

**Table 3:** Comparison of frequency of additional drug resistance in MDR and non-MDR TB patients at the Korle-Bu Teaching Hospital Chest Clinic, Accra

Characteristic	Non-MDR-TB (*N* = 81)	MDR-TB (*N* = 31)	*P* value
Streptomycin resistance			< 0.001
Yes	20 (24.7%)	23 (74.2%)	
No	61 (75.3%)	8 (25.8%)	
Ethambutol resistance			< 0.001
Yes	1 (1.2%)	7 (22.6%)	
No	80 (98.8%)	24 (77.4%)	

*MDR-TB* multidrug resistant tuberculosis

## Discussion

The main findings of this study are the high prevalence of streptomycin resistance and MDR-TB among previously treated TB patients, as well as a high prevalence of pre-XDR-TB among the MDR-TB patients. In our study 28% of the patients had MDR-TB. In addition, 36% of the MDR-TB patients had resistance to either a quinolone or second-line aminoglycoside (pre-XDR TB), and would have a suboptimal response to the standardized MDR-TB regimen given to all patients with MDR.

The frequency of MDR-TB in the current study is similar to the 24% reported in a previous study in Ghana that evaluated 17 patients [9], but it is lower than the 36% that we reported among 28 chronic smear-positive pulmonary TB cases who had failed one to three supervised retreatment regimen [12]. The higher rate that we previously observed among the chronic cases may be due to the repeated use of the Category II regimen in patients who had failed or relapsed on a previous retreatment since MDR-TB could not be diagnosed at a time when DST was not available at our center. Other studies from the West African region, one in Cameroon [13], and another in Nigeria [14] reported MDR-TB prevalence among smear positive previously treated patients of 12% and 19%, respectively. These rates are lower than the rates of the 24 to 36% in the Ghanaian studies [9, 12]. In contrast a study in Burkina Faso reported the frequency of MDR-TB to be 50.5% among previously treated patients [15]. These variable trends between countries in the West African region underscore the need for each country to carefully examine their local epidemic and tailor national treatment guidelines to the local situation. It is yet to be determined if the standardized regimen for MDR-TB in Ghana is effective.

This study confirms the high prevalence of streptomycin resistance among previously treated TB patients in Ghana as reported in other Ghanaian studies [9, 12]. An explanation for the high levels of streptomycin resistance could be because of the many years of streptomycin use for first-line treatment up until 2005–2007, when ethambutol gradually replaced streptomycin in the standard regimen. Some streptomycin resistance could also have resulted from reactivation of latent TB infection resulting from transmission of primary streptomycin resistance present in the community. A large survey across Ghana of 2064 new smear positive cases from 2001 to 2004 found that 17% of cases had streptomycin resistance [16].

The continuing use of streptomycin for Category II regimens since the study in the early 2000’s might have amplified the presence of streptomycin resistance, as in our previous study which found 80% streptomycin resistance among previously treated chronic TB patients [9, 12]. These findings suggest that the high level of streptomycin resistance presently among previously treated patients in Ghana will render ineffective the standardized Category II regimen that adds only streptomycin to the first-line therapy. A study by Nakanwagi-Mukwaya and colleagues in Uganda have shown that while only 32% of previously treated patients received Category II treatment there were no differences in outcome with those on Category I [5]. Another study in Peru found 20% resistance to streptomycin and found outcomes were better with Category I than Category II treatment, in support of the phasing out of Category II treatment in Peru [17]. Additionally in Thailand, patients with rifampicin resistance or MDR-TB who had received Category II treatment were found to have poorer outcomes [18].

In Ghana about 15,000 patients are diagnosed with TB annually, and the number of previously treated patients presenting for retreatment make up 5–6% of all TB patients diagnosed. In a review of national TB control programme (NTP) data on previously treated patients in 2015, 46% were relapse patients, 26% had defaulted or failed treatment, and 27% were grouped together and included extrapulmonary TB, smear negative pulmonary TB, and patients who were unable to do a sputum smear. There was an average treatment success rate of 77% across all categories of previously treated patients, and a mean mortality of 11%. At the Korle Bu Teaching Hospital (KBTH) 216 previously treated smear positive patients, registered from January 2010 to June 2015, were found to have a low treatment success rate of 59%, and 17% who relapsed/failed treatment (T. Bouton, A. Forson unpublished data). Other outcomes were 11% transferred out (to other centres), while default was 4%. NTP data from 2015 (Table 4) showed differences between the regions, with Ashanti and Western Regions having the highest numbers of previously treated TB patients, yet with the highest treatment success rates (85–90%), and Greater Accra Region which had treatment success rates of 80%. However, the two largest teaching hospitals in the southern sector (Korle Bu Teaching Hospital in Accra) and the northern sector (Komfo Anokye Teaching Hospital in Ashanti) had the poorest outcomes, with mortality rates of 35% and 42% respectively. High mortalities are the main reason for low success rates in these two facilities, which provide in-patient care for the more severely ill patients.

**Table 4:** Summary of previously treated TB patients in Ghana in 2015, by region

Region	Relapse	Failure	Default	Other	TOTAL
ASHANTI	110	21	26	26	183
WESTERN	55	10	22	22	109
CENTRAL	42	7	10	10	69
EASTERN	43	12	16	16	87
G-ACCRA	54	13	18	18	103
NORTHERN	10	9	5	5	29
U-EAST	17	16	5	5	43
U-WEST	11	7	3	3	24
VOLTA	22	14	8	8	52
BAR^a^	55	15	6	6	82
KATH^a^	10	0	2	2	14
KBTH^a^	13	3	6	6	28
TTH^a^	3	0	0	0	3
Grand Total	445	127	127	268	967

^a^*BAR* Brong Ahafo Region, *KATH* Komfo Anokye Teaching Hospital

*KBTH* Korle-Bu Teaching Hospital, *TTH* Tamale Teaching Hospital

Similar drug resistance surveillance studies are necessary in other regions, particularly in the Northern sector, as this study’s findings cannot be said to be representative of the whole of Ghana. TB treatment outcomes in the country to a large extent depend on drug sensitivity patterns in the region, as well as the effectiveness of DOTS supervision and monitoring. Differences between the regions may exist, as in the Ashanti region (in the Northern half of Ghana) where in spite of high treatment relapse/failure patient numbers they had one of the highest retreatment success rates (85.0%) on the Category II regimen. Using the tertiary hospital in this study, providing care for the more severely ill patients, could have predisposed to the selection for culture of the more resistant isolates, which would be expected to be the cause of more severe disease.

In the current study, 36% of the MDR-TB isolates were pre-XDR. A previous study that included only 17 previously-treated cases, identified that two (12%) of the MDR-TB cases were pre-XDR [9]. The presence of pre-XDR-TB in patients that are naïve to second-line therapy in the current and the prior Ghana studies is surprising, and could be related to antimicrobial resistance (ciprofloxacin, gentamycin, amikacin) as a result of the frequent use of these for other infectious diseases [19].

## Conclusion

Overall, our findings suggest that first-line and second-line DST is essential to aid the design of effective regimens for these groups of patients in Ghana. The present study underscores the need for a scale up of routine DST in previously treated TB patients and the use of effective regimens based on susceptibility test results, in order to avoid the emergence of XDR-TB in Ghana. This situation is not unique to Ghana. Ongoing use of standardized regimens for previously treated TB patients without DST guidance may lead to amplification and transmission of resistant TB.

Limitations of our study include the fact that our sample size was small and was not representative of the whole country. Our study represented mainly the region of the capital, Greater Accra, and regions in the southern part of Ghana, and precludes generalization to the rest of Ghana. Another limitation of this study was that only previously treated TB patients with a positive sputum smear sample were selected, as routine culture of all previously treated patients was not available at the time of the study. Currently, all previously treated tuberculosis patients have sputum cultures done, so there is the need in subsequent studies to include all culture positive patients. Further studies and review of nationwide drug resistance surveillance data will be necessary.

## Abbreviations

AFB: Acid fast bacilli
DST: Drug sensitivity testing
EDCTP: The European & Developing Countries Clinical Trials
HR: Isoniazid, rifampicin
HRE: Isoniazid, rifampicin, ethambutol
HRZE: Isoniazid, rifampicin, parazinamide, ethambutol
MDR: Multi-drug resistant
MDR-TB: Multi-drug resistant tuberculosis
MGIT 960: Mycobacteria growth indicator tube (MGIT) 960 system
MGIT MTBcID: MGIT Mycobacteria tuberculosis identification test (Becton, Dickinson and Company)
MRC, Gambia: Medical Research Council Unit, the Gambia
pre-XDR: Pre-extensively drug resistant
pre-XDR-TB: Pre-extensively drug resistant tuberculosis
SHRZE: Streptomycin, isoniazid, rifampicin, parazinamide, ethambutol
TB: Tuberculosis
XDR-TB: Extensively drug resistant tuberculosis
